# Freestanding rGO-SWNT-STN Composite Film as an Anode for Li Ion Batteries with High Energy and Power Densities

**DOI:** 10.3390/nano5042380

**Published:** 2015-12-18

**Authors:** Taeseup Song, Junghyun Choi, Ungyu Paik

**Affiliations:** 1 School of Materials Science and Engineering, Yeungnam University, Gyeongsan 712-749, Korea; E-Mail: tsong@yu.ac.kr; 2 Department of Energy Engineering, Hanyang University, Seoul 133-791, Korea; E-Mail: junghchoi@hanyang.ac.kr

**Keywords:** Si-Ti-Ni alloy, graphene, carbon nanotubes, freestanding electrode, Li ion batteries

## Abstract

Freestanding Si-Ti-Ni alloy particles/reduced graphene oxide/single wall carbon nanotube composites have been prepared as an anode for lithium ion batteries via a simple filtration method. This composite electrode showed a 9% increase in reversible capacity, a two-fold higher cycle retention at 50 cycles and a two-fold higher rate capability at 2 *C* compared to pristine Si-Ti-Ni (STN) alloy electrodes. These improvements were attributed to the suppression of the pulverization of the STN active material by the excellent mechanical properties of the reduced graphene oxide-single wall carbon nanotube networks and the enhanced kinetics associated with both electron and Li ion transport.

## 1. Introduction

Significant efforts have been devoted towards using Si materials for high energy density Li ion batteries (LIBs) due to the high theoretical capacity and relatively low working potential of Si [1]. However, a fast capacity fading, caused by the large volumetric change of Si during cycling, and the poor rate capability resulting from the sluggish kinetics associated with Li ion and electron transport, limits its practical use [2,3]. Various strategies for engineering Si-based electrode configurations by manipulating the dimensions, geometry, and composition have been explored to overcome these problems. Previous works have demonstrated that utilizing nano-sized Si materials, including nanoparticles [4], thin films [5], and nanowires [6], are effective for improving the cycle performance due to a relatively smaller absolute volume change and facile stress relaxation during cycling, compared to that of bulk Si [4,5,6]. Porous Si materials, such as Si nanotubes [7], double-walled silicon nanotubes [8], and a Si inverse opal structure [9], have also been extensively explored. Free space in these porous structures and their high surface area improved the electrochemical properties due to the facile accommodation of the large volume change associated with Li ions and the enhanced Li ion kinetics [10]. Although nanostructured Si materials showed promise in improving the electrochemical properties, their practical use in commercialized LIBs has been limited due to their high cost, significant undesirable side reactions caused by the large surface area, and the low volumetric capacity resulting from the low tap density [11].

Recently, a Si-Ti-Ni (STN) alloy has received significant attention as a promising Si-based anode material for commercial applications. The nano-sized Si particles were incorporated into the micro-sized nickel-titanium (Ni-Ti) alloy matrix, which was inactive to lithium and has highly elastic characteristics. These physicochemical properties of the STN alloy enable stable cycle performance resulting from (i) effective accommodation of the volume change of embedded Si nanoparticles in the Ti-Ni alloy matrix, (ii) suppression of the side reaction by preventing direct contact between the Si and the electrolyte, and (iii) high tap density due to the large STN particles. However, the native oxides formed on the STN alloy surface, including SiO*_x_*, TiO*_x_* and NiO*_x_*, cause degraded electrochemical properties, including poor rate capability and low initial coulombic efficiency [12,13]. In a previous study, we demonstrated that a nitridated STN alloy electrode exhibited improvements in rate capability, cycle retention, and reversible capacity due to the enhanced electrical conductivity and mechanical strength caused by the nitride compounds formed on the STN surface [14]. However, the improvement in the electrochemical properties of the nitridated STN electrode was limited due to the use of insulating binders and the localized contact points between the STN particles for electron transport, which limited the free space in the electrode available to accommodate the volume change [15,16]. Furthermore, the necessity of a heavy current collector for electrode preparation induced a significant decrease in the energy density [17]. Further improvements in the electrochemical properties of STN electrodes were necessary for practical use.

**Figure 1:**
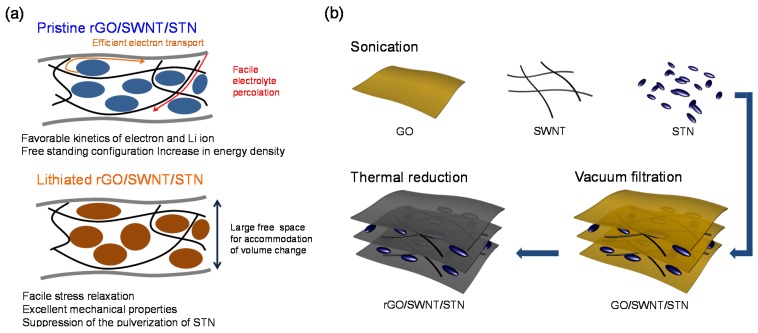
Schematic illustration of (**a**) advantages of freestanding reduced graphene oxide /single wall carbon nanotube/Si-Ti-Ni (rGO-SWNT-STN) composite film and (**b**) its fabrication process.

Here, we report freestanding reduced graphene oxide (rGO)/single wall carbon nanotube (SWNT)/STN particle composites, prepared by a simple vacuum filtration method, as anodes for high power and energy densities LIBs with robust performance. Graphene has been extensively explored as an electrode component for rechargeable energy storage devices [18,19]. Its two-dimensional geometry, high electrical conductivity, and excellent mechanical properties enable the stable dispersion of STN particles, the enhancement of electrical conductivity over the electrode, and suppression of STN particle pulverization during cycling, leading to significant improvements in electrochemical properties of STN electrodes [20]. The SWNTs were employed in composite films to improve Li ion kinetics by enabling better electrolyte percolation into the composite film [21]. The excellent mechanical and electrical properties of the composite film originating from the rGO and SWNT networks allowed a freestanding electrode configuration without a metal current collector [21,22]. Our designed electrode configuration provided various advantages (Figure 1a) including: (i) improvement in the cycle performance by facile stress relaxation in the longitudinal direction, (ii) improvement in the rate capability by enhancing the kinetics associated with Li ion and electron transport, and (iii) an increase in the energy density due to the removal of the heavy metal current collector. Significant improvement in electrochemical performance, including reversible capacity, cycle retention, rate capability, and cycle performance, was achieved by employing the freestanding electrode configuration.

## 2. Results and Discussion

Figure 1b shows the fabrication process used for the preparation of the freestanding rGO-SWNT-STN composite film. GO, SWNT, and STN particles were homogenously dispersed in the sodium dodecyl sulfate (SDS)-containing aqueous solvent using sonication. The freestanding GO-SWNT-STN composite film was readily prepared via a typical vacuum filtration method. The rGO-SWNT-STN composite film was obtained by the reduction of GO in the composite film at 300 °C under a N_2_ atmosphere. Two-dimensional rGO sheets provided efficient electron transport pathways and prevented the pulverization of STN active materials in the composite. SWNTs in the composite further enhanced the mechanical properties and electrical conductivity of the composite. In addition, the presence of SWNTs in the composite enabled facile electrolyte penetration into the composite resulting in reduced agglomeration of STN particles [21].

**Figure 2:**
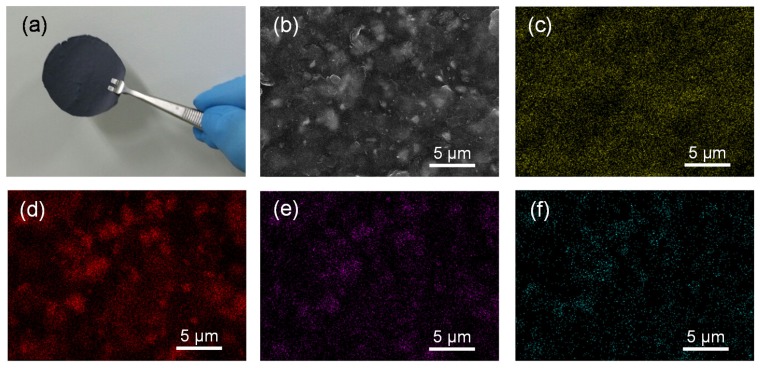
(**a**) Photograph and (**b**) field emission scanning electron microscopy (FE-SEM) image of freestanding GO-SWNT-STN composite film. Elementary mapping images of (**c**) carbon, (**d**) silicon, (**e**) titanium, and (**f**) nickel in the composite film.

Figure 2 shows a photograph, an SEM image, and elementary mapping images for GO-SWNT-STN composites. As shown in Figure 2a, flexible and freestanding GO-SWNT-STN composite films were successfully prepared. The pristine STN powder had micro-sized diameter particles ranging from 1 µm to 10 µm. It was difficult to obtain a clear SEM image due to the charging effect caused by the low electrical conductivity of the GO-SWNT-STN composite film. The spatial distribution of C, Si, Ti, and Ni elements in the composite were analyzed using energy dispersive X-ray spectrometry (EDS) elemental mapping (Figure 2c–f). As shown, Si, Ti, and Ni signals originated from STN particles, and the C signal originated in the GO and SWNT. The intensity difference in the element signals was attributed to the different atomic ratios of these elements in the composite film. All elemental signals were uniformly distributed in the overall composite film, which suggested that STN particles were homogeneously embedded in the GO/SWNT matrix without agglomeration. This property was critical to achieving stable cycle performance and battery safety by controlling the volume expansion behavior of the electrode [18].

**Figure 3:**
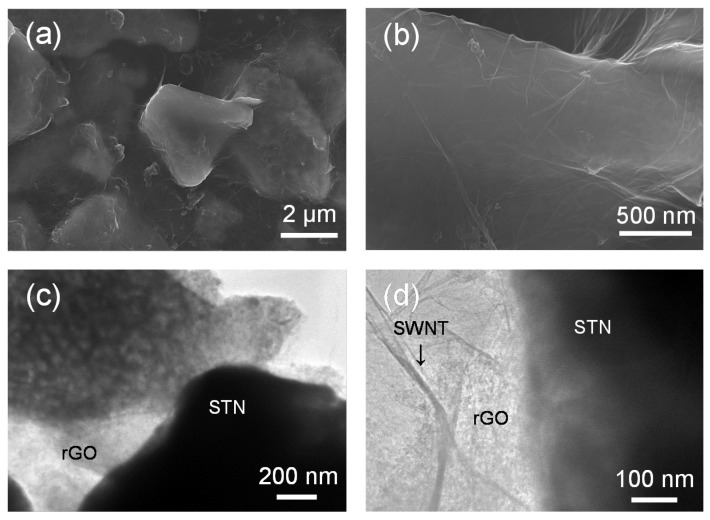
(**a**) SEM and (**b**) transmission electron microscope (TEM) images of rGO-SWNT-STN composite film.

The morphology and microstructure of the rGO-SWNT-STN freestanding film was characterized using electron microscopy. Figure 3a,b is low-magnification and high-magnification SEM images of the rGO-SWNT-STN composite film, respectively. Very clear SEM images were obtained compared to that of the GO-SWNT-STN composite film due to the improved electrical conductivity of the composite film after the reduction of GO. We clearly observed that the STN particles were fully embedded in the rGO-SWNT composite networks. A good network geometry in the composite films is essential for improving both the mechanical and electrical properties. The wrinkled morphology on the surface of the STN particle was attributed to wrapping of rGO sheets around the particles (Figure 2b). Because of the intimate contact generated by this wrapping behavior, electrons could easily move along the rGO sheet on the surfaces of the STN particles. The microstructures of STN particles and rGO-SWNT-STN composite films were further characterized using a transmission electron microscope (TEM), as shown in Figure 3c,d. The different scattering abilities of each metal in the STN particle induced noticeable differences in the brightness between the Ti-Ni alloy matrix and Si domains, Figure 3c. Relatively dark and bright regions represent the Ni-Ti matrix and Si domains, respectively. It is clearly observed that STN particles formed a good network through the rGO sheet. High-magnification TEM images indicated the good dispersion of SWNTs in the composite film. SWNTs were anchored on the surfaces of both rGO and STN particles, which suggested that SWNTs could provide a continuous and effective electron pathway in the freestanding composite film. The strong mechanical properties of the rGO-SWNT networks were able to resist the large stresses caused by the large volumetric change associated with Li, leading to robust cycle performance [23].

**Figure 4:**
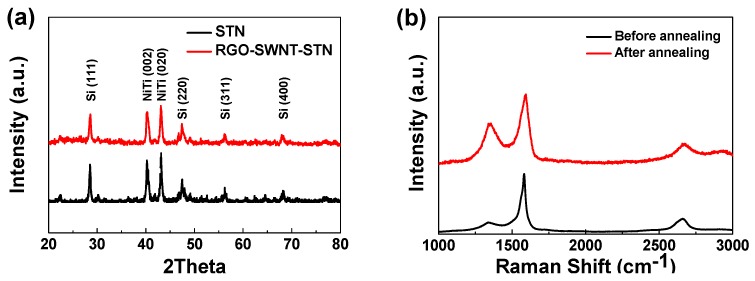
(**a**) X-ray diffraction patterns of Si-Ti-Ni (STN) and rGO-SWNT-STN, (**b**) Raman spectra of freestanding films, before and after annealing.

An X-ray diffractometer (XRD) and Raman spectroscopy were employed to investigate the effect of thermal reduction process on the physicochemical properties of the freestanding composite film. XRD results (Figure 4a) showed high intensity peaks related to the crystalline Si and Ni-Ti alloy. However, no noticeable change in the crystal phase was observed, which suggests that the mild annealing process did not induce crystallographic ordering of the STN particle. As shown in Figure 4b, two distinguishable peaks at 1588 cm^−1^ and 1345 cm^−1^, corresponding to a graphite band (G band) and a disorder band (D band), respectively, were observed in both composite films. The D band and G band are associated with a disorder induced phonon mode and defects/grain boundaries, respectively [24]. The rGO-SWNT-STN composite film exhibited higher D to G intensity ratios (*I*_D/G_ ~ 0.79) compared to that of the GO-SWNT-STN composite film (*I*_D/G_ ~ 0.20), which implies the successful reduction to rGO from GO [25]. The peak shift of the 2D band from 2661 cm^−1^ to 2671 cm^−1^ also provided evidence for the reduction of GO [26].

**Figure 5:**
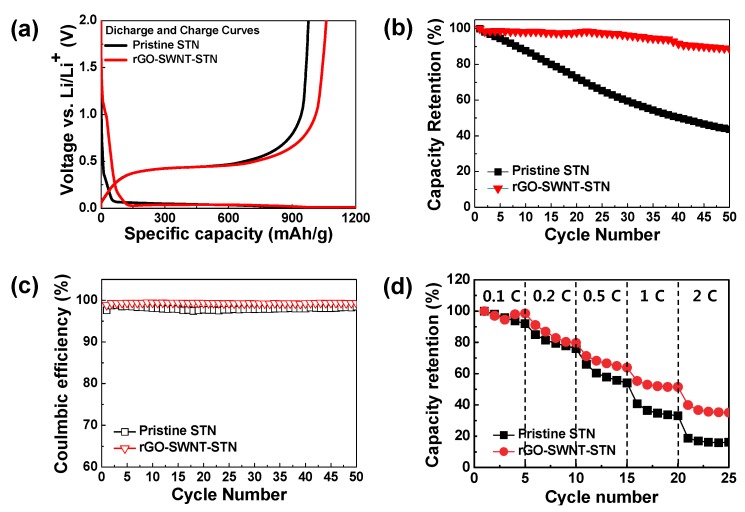
Electrochemical performances of the pristine STN and the rGO-SWNT-STN electrodes. (**a**) Initial cycle voltage profiles at a rate of 0.1 *C*, (**b**) Cycle retentions at a rate of 1 *C*, (**c**) Coulombic efficiency at a rate of 1 *C*, (**d**) Rate capabilities at various *C* rates.

**Figure 6:**
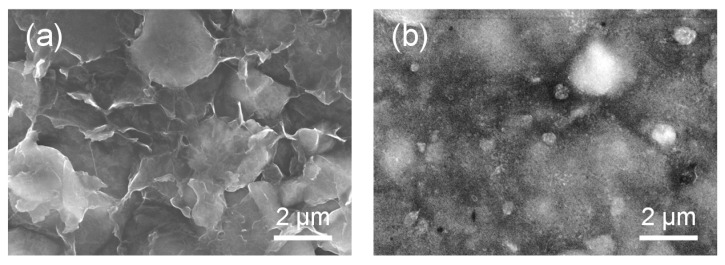
SEM images of rGO-SWNT-STN electrode for (**a**) full lithiation, and (**b**) delithiation at the first cycle.

**Figure 7:**
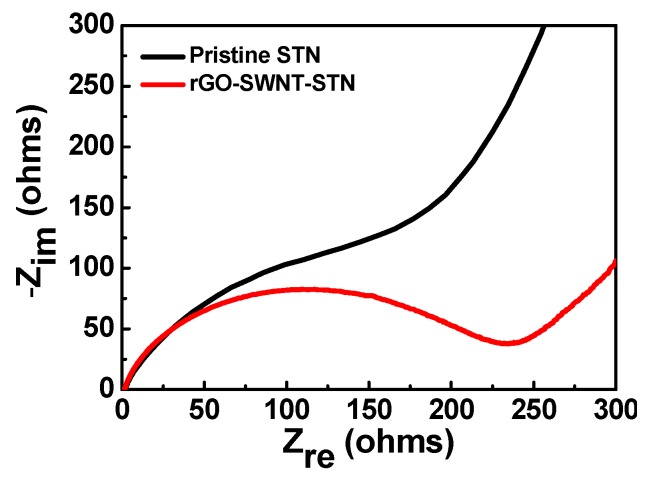
Electrochemical impedance spectra of the pristine STN and the rGO-SWNT-STN electrodes.

The electrochemical properties of the pristine STN electrode prepared on the Cu current collector, and freestanding rGO-SWNT-STN composite electrode were evaluated in the voltage range of 0.01 and 2.0 V (*vs*. Li/Li^+^) (Figure 5). Both electrodes showed voltage plateaus around 0.1 V, which corresponded to the alloy reaction of the crystalline Si with lithium [7]. The small plateau around 1 V, observed in the freestanding rGO-SWNT-STN composite electrode, was attributed to the formation of a solid electrolyte interface (SEI) layer [27]. The pristine STN electrode exhibited initial discharge (lithiation) and charge (delithiation) capacities of 1046 mAh/g and 976 mAh/g, respectively. On the other hand, the rGO-SWNT-STN composite electrode delivered higher discharge and charge capacities of 1215 mAh/g and 1061 mAh/g, respectively. Although the rGO-SWNT-STN composite electrode exhibited a slightly lower initial coulombic efficiency of 87.3% compared to that of the pristine STN electrode (93.3%) due to the formation of an SEI layer, it delivered a much higher reversible capacity. The increased reversible capacity of the composite film was attributed to the enhancement of the electrical conductivity via the rGO-SWNT networks, as well as the improvement in Li ion kinetics via the facile electrolyte percolation due to the SWNTs. Within three cycles, the rGO-SWNT-STN composite electrode approached coulombic efficiencies of more than 99%, and it showed higher coulombic efficiencies compared to those of the pristine STN electrode after the first cycle (Figure 5c), implying improved electrochemical reversibility. The cycle performances were evaluated at a current rate of 1 *C* for 50 cycles (Figure 5b). Pre-cycling was conducted to form a stable SEI layer at 0.1 *C* for three cycles before monitoring the cycle performances. The pristine STN electrode and freestanding rGO-SWNT-STN composite electrode showed capacity retentions of 43.70% and 88.97% at 50 cycles, respectively. The enhanced cycle performance of the rGO-SWNT-STN composite electrode was attributed to the improved mechanics associated with cycling. The freestanding electrode configuration allowed facile accommodation of the large volume change in the longitudinal direction. Furthermore, the excellent mechanical properties of the rGO-SWNT networks prevented pulverization of the STN active material. We carefully monitored the morphological changes of the rGO-SWNT-STN composite electrode after full lithiation and delithiation in the first cycle (Figure 6). After full lithiation, the composite electrode maintained its networked structure without noticeable mechanical degradation, such as cracking or pulverization. As shown in Figure 6b, mechanical degradation was not observed in the composite electrode, even after severe volume changes, which implies that the freestanding electrode configuration improved the mechanical properties leading to improvement in the electrochemical properties. Figure 5d shows the rate capabilities of both electrodes. The rGO-SWNT-STN composite electrode showed excellent rate capability compared to that of the STN electrode, especially at high *C* rates. For example, the rGO-SWNT-STN composite electrode showed a capacity retention of 35.07% at a rate of 2 *C*. On the other hand, the STN electrode exhibited a capacity retention of 16.18% at the same current rate. The improved rate capability of the rGO-SWNT-STN composite electrode was attributed to the enhancement of the kinetics associated with the electron and Li ion kinetics by rGO and SWNT networks. Electrochemical impedance spectra (EIS) for the STN electrode and rGO-SWNT-STN composite electrode also indicated an enhancement in the kinetics (Figure 7). Ohmic resistances were 2.2 and 1.7 Ω for the pristine and the rGO-SWNT-STN electrode, respectively. The rGO-SWNT-STN electrode showed a smaller semicircle, which corresponded to the polarization resistance (*i.e.*, the charge-transfer resistance). Our results imply that electrode configuration engineering along with composition engineering enables significant improvements in the electrochemical properties.

## 3. Experimental Section

### 3.1. Preparation of Freestanding rGO-SWNT-STN Film

A SNT powder with an atomic ratio of 66% (Si), 17% (Ti), 17% (Ni) (MK Electron, Yongin, Korea) having a particle size distribution from 1 μm to 10 μm was used as a starting material. First, 0.004 g of SWNT, 0.004 g of graphene oxide, and 0.032 g of STN powder were dispersed in a 5 wt % sodium dodecyl sulfate (SDS, ACS Reagent, Sigma Aldrich, St. Louis, Missouri, USA) aqueous solution. Tip sonication was conducted to disperse starting materials for 20 min in an ice bath. The solution was filtered and washed with distilled water several times to remove residual SDS. After drying in a vacuum oven at 60 °C for 24 h, a freestanding film was obtained. Finally, a freestanding film was annealed at 300 °C for 1 h in a N_2_ atmosphere to reduce the graphene oxide.

### 3.2. Characterization

The morphology of the rGO-SWNT-STN film was characterized by field emission scanning electron microscopy (FESEM, JSM-7600F, JEOL, Tokyo, Japan) and transmission electron microscopy (TEM, JEM-2010, JEOL, Tokyo, Japan). The chemical composition was observed using scanning electron microscopy (SEM, Nova NanoSEM, FEI, Hillsboro, OR, USA) equipped with energy dispersive X-ray spectroscopy (EDS). An X-ray diffractometer (XRD, D/MAX RINT-2000, Rigaku, Tokyo, Japan) was used to investigate crystallographic structure. Raman spectra were measured to confirm reduction of graphene oxide using Raman spectroscopy (NRS-3100, JASCO, Easton, MD, USA).

### 3.3. Measurement of Electrochemical Performance

Coin type half cells (2032R type) were fabricated to evaluate the electrochemical performances of rGO-SWNT-STN films. Pure lithium metal foils served as a counter electrode, and 1.3 M LiPF_6_ in ethylene carbonate-diethylene carbonate (EC-DEC, 3:7 vol %, PANAX) was used as an electrolyte. The cells were assembled in an Ar-filled grove box. The coin cells were tested using a battery cycle tester (TOSCAT 3100, Toyo Systems, Iwaki, Japan). Electrochemical impedance spectroscopy (EIS) was measured in the frequency range of 250 kHz to 10 mHz at amplitude of 5 mV using a potentiostat (PARSTAT MC, Princeton Applied Research, Oak Ridge, TN, USA).

## 4. Conclusions

A freestanding rGO-SWNT-STN composite was successfully prepared as an anode material via a facile filtration process, and its electrochemical properties were evaluated. The freestanding electrode configuration and the rGO-SWNT networks accommodated the large volume change, provided excellent mechanical properties, and showed favorable lithium ion and electron transport kinetics. These properties led to significant improvements in the electrochemical performance. The freestanding rGO-SWNT-STN composite electrode showed an increase in reversible capacity, a two-fold higher cycle retention at 50 cycles, and a two-fold higher rate capability at 2 *C* compared to those of pristine STN electrodes.
